# Analysis of the Effects of Food Additives on *Porphyromonas gingivalis*

**DOI:** 10.3390/pathogens11010065

**Published:** 2022-01-04

**Authors:** Mai Shinohara, Miki Maetani, Chiharu Kitada, Yasuko Nishigami, Ayaka Yazawa, Shigeki Kamitani

**Affiliations:** Nutrition Support Course, Graduate School of Comprehensive Rehabilitation, Osaka Prefecture University, Habikino 583-8555, Japan; vm9.st.l0@gmail.com (M.S.); 3428hsm@gmail.com (M.M.); hamamotochiharu11@gmail.com (C.K.); n.ysk0706@outlook.jp (Y.N.); ayazawa@rehab.osakafu-u.ac.jp (A.Y.)

**Keywords:** periodontal pathogens, food additives

## Abstract

This study aims to investigate six food additives (octanoic acid, decanoic acid, acesulfame K, aspartame, saccharin, and sucralose) used in foods for the elderly or people with dysphagia because of the effect of these food additives on *Porphyromonas gingivalis* (*P. gingivalis*), which is a keystone pathogen of periodontal diseases. The growth of *P. gingivalis* was inhibited by 5 mM octanoic acid, 1.25 mM decanoic acid, 1.25% acesulfame K, 0.0625% aspartame, 0.03125% saccharin, and 0.625% sucralose. In addition, these food additives showed bactericidal activity for planktonic *P. gingivalis* (5 mM octanoic acid, 5 mM decanoic acid, 0.25% aspartame, 0.25% saccharin, and 5% sucralose). Moreover, biofilm formation was inhibited by 10 mM octanoic acid, 10 mM decanoic acid, 10% acesulfame K, 0.35% aspartame, 0.5% saccharin, and 7.5% sucralose. Moreover, the same concentration of these food additives without aspartame killed *P. gingivalis* in the biofilm. Aspartame and sucralose did not show cytotoxicity to human cell lines at concentrations that affected *P. gingivalis.* These findings may be useful in clarifying the effects of food additives on periodontopathogenic bacteria.

## 1. Introduction

Periodontal disease is an inflammatory disease caused by infection with periodontopathogenic bacteria [1,2,3]. It is a disease of the gingiva and periodontal tissues. The long-term presence of periodontopathogenic bacteria in the oral cavity causes inflammation of the gingiva and further destruction of periodontal tissue.

The prevalence of periodontal disease is about 45% to 50% of adults in the mild form and becomes more than 60% in over 65-aged people. Severe periodontitis is estimated to affect 11.2% of the world’s adult population [4]. There are three risk factors for periodontal disease: environmental factors, such as smoking, nutrition, and alcohol consumption; host factors, such as diabetes and aging; and bacterial factors, such as periodontopathogenic bacteria. In recent years, a relationship between periodontopathogenic bacteria and various systemic diseases, such as aspiration pneumonia, osteoporosis, and cardiovascular diseases, has been strongly suggested [5,6,7,8,9,10,11,12].

According to the expanded human oral microbiome database, there are more than 700 kinds of bacteria that live in the oral cavity [13] (http://www.homd.org, accessed on 11 November 2021). *Porphyromonas gingivalis* (*P. gingivalis*) is frequently detected in deep periodontal pockets and is considered the most critical bacterium in periodontal disease [14]. *P. gingivalis* is a Gram-negative anaerobic asaccharolytic bacterium and possesses a variety of virulent factors, such as cysteine proteinases (gingipains), lipopolysaccharide (LPS), hemagglutinins, and adhesin as fimbriae [15]. In addition, studies using mouse models of periodontal disease induced by *P. gingivalis* infection have shown that *P. gingivalis* is a keystone pathogen that causes dysbiosis by disrupting the homeostasis of the indigenous bacterial flora with the host through complementation [16]. It is now clear that such dysbiosis of the oral microflora affects not only diseases specific to the oral cavity, such as dental caries and periodontal disease, but also organs and tissues other than the oral cavity [2]. In other words, the transition from host-microbe symbiosis to dysbiosis is regarded as susceptibility to periodontitis. The oral cavity is influenced by aging. Oral lesions were significantly dominant in mature and elderly age groups compared to the groups of children and adolescents [17] and salivary hypofunction (reduced saliva flow rate) was found in the vulnerable elderly [18]. Bacterial composition was also more diverse in youth microbiomes when compared to adults [19]. Therefore, changes in the oral environment are thought to affect the oral microbiome.

Prevention is as important as treatment in the treatment of periodontal disease [20]. It is crucial to inhibit the growth of periodontopathogenic bacteria and the production of virulence factors, and attention to oral hygiene and food choices have a significant impact on prevention in order to prevent periodontal disease [21]. Foods high in carbohydrates, which adhere to the oral cavity and serve as nutrients for bacteria, exacerbate periodontal disease [22,23], but the details of many foods are unknown. There have been reports that artificial sweeteners have antibacterial effects on *P. gingivalis* and *A. actinomycetemcomitans* [24]. However, the effects of food additives in foods on periodontal disease are largely unknown. Many food additives are artificially produced and are also used in swallowing adjustment foods for people with dysphagia (Nestle Health Science, Pepatamen, <https://www.nestlehealthscience-me.com/en/brands/peptamen/peptamen>. [accessed 11 November 2021]).

In this study, we aimed to clarify the effects of food additives on the pathogenicity of *P. gingivalis* as one of the periodontopathogenic bacteria. We examined six food additives used in foods, especially those developed for the elderly or used in swallowing foods for people who have difficulty swallowing, on periodontal bacteria, including bacterial growth and biofilm formation.

## 2. Results

### 2.1. Growth Inhibition Effect of Food Additives

**Figure 1:**
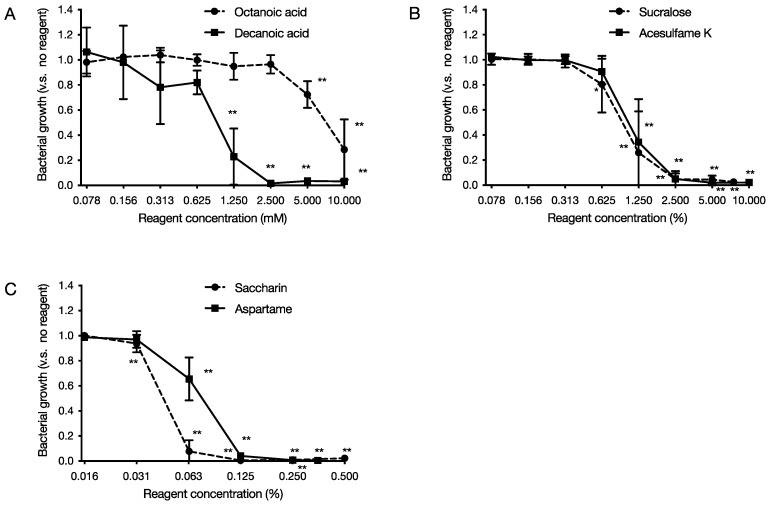
Effect of food additives on the growth of *P. gingivalis.* After 48 h of incubation at 37 °C, under anaerobic conditions, turbidity was measured. The graph’s vertical axis shows the cell growth rate when the cell growth rate in the absence of food additives is 1.0, and the horizontal axis shows the concentration of food additives added. (**A**) Medium-chain fatty acids (octanoic acid and decanoic acid), (**B**,**C**) Artificial sweeteners ((**B**) acesulfame K and sucralose, (**C**) aspartame and saccharin). Data in the graphs represent the mean ± SD. n = 6, representative data from more than ten repeated experiments are shown. *, ** indicates that each is significant at *p* < 0.05, 0.01, which is significant for the bacterial growth rate without food additives.

Six food additives, octanoic acid and decanoic acid (medium-chain fatty acids) and artificial sweeteners, acesulfame K, aspartame, saccharin, and sucralose, were tested for growth inhibition against *P. gingivalis*. After 48 h of incubation under anaerobic conditions at 37 °C, the turbidity of the culture medium was measured. The results showed that medium-chain fatty acids inhibited growth in a concentration-dependent manner, starting from 5 mM for octanoic acid and 1.25 mM for decanoic acid (Figure 1A). As for the artificial sweeteners, acesulfame K inhibited growth in a concentration-dependent manner from 1.25%, with aspartame from 0.0625%, saccharin from 0.0625%, and sucralose from 1.25% (Figure 1B,C). The MICs of octanoic acid were more than 10 mM, decanoic acid 2.5 mM, acesulfame K 5%, aspartame 0.25%, saccharin 0.125%, and sucralose 7.5%. Other more virulent strains of *P. gingivalis,* including W83 and TDC60, showed the same sensitivity to growth inhibition by these six food additives (Figure S1).

### 2.2. Bactericidal Activity of Food Additives

**Figure 2:**
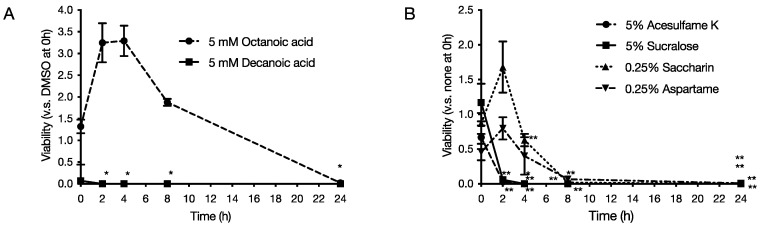
Effect of food additives on survival of *P. gingivalis.* Food additives were added to *P. gingivalis* prepared to 1 × 10^8^ CFU/mL and allowed to act for each hour at 37 °C under anaerobic conditions. After the action, the bacteria were applied to an agar medium and cultured for 10 days, and the number of viable bacteria was measured. The graph’s vertical axis shows the survival rate when the survival rate in the absence of food additives was set at 1.0, and the horizontal axis shows the incubation time. (**A**) 5 mM octanoic acid and 5 mM decanoic acid, (**B**) 5% acesulfame K, 5% sucralose, 0.25% saccharin, and 0.25% aspartame. Each data in the graph represents the mean ± SD. Data from n = 3 experiments are shown. *, ** indicates that each is significant at *p* < 0.05, 0.01, which is significant relative to survival in the absence of food additives.

Each food additive was added to the culture solution of *P. gingivalis* and allowed to act for 2, 4, 8, and 24 h at 37 °C under anaerobic conditions. The final concentrations of each food additive were 5 mM octanoic acid and 5 mM decanoic acid for medium-chain fatty acids, and 5% acesulfame K, 0.25% aspartame, 0.25% saccharin, and 5% sucralose for artificial sweeteners. Bacteria after the action were collected and cultured for 10 days, and the number of viable bacteria was measured by the number of bacterial colonies formed. As a result, it was clarified that decanoic acid reduced the viable cell count to 0 after 2 h of culture for medium-chain fatty acids (Figure 2A). As for artificial sweeteners, it was revealed that acesulfame K and sucralose reduced the viable cell count to 0 after 2 h of culture (Figure 2B).

### 2.3. Inhibitory Effect of Food Additives on Biofilm Formation

**Figure 3:**
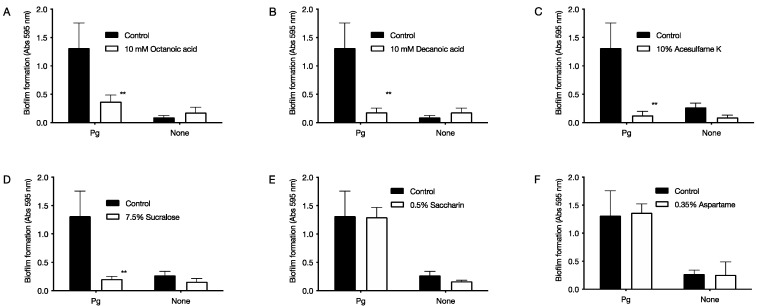
Effect of food additives on survival of *P. gingivalis.* Biofilms were formed under anaerobic conditions at 37 °C after seeding *P. gingivalis* with 1 × 10^9^ CFU/mL of food additives. The biofilm was stained with crystal violet staining solution to determine the biofilm formation ability. The graph’s vertical axis shows the absorbance value at 595 nm (amount of biofilm formed). The horizontal axis Pg shows the data with *P. gingivalis*. None shows data without *P. gingivalis*, and the control shows data without food additives. (**A**) 10 mM octanoic acid, (**B**) 10 mM decanoic acid, (**C**) 10% acesulfame K, (**D**) 7.5% sucralose, (**E**) 0.5% saccharin, and (**F**) 0.35% aspartame. Data in the graph represent the mean ± SD. n = 6, representative data from more than ten repeated experiments. ** indicates *p* < 0.01, which is significant for the amount of biofilm formation in the absence of food additives.

Each food additive was added to the culture solution of *P. gingivalis,* and a biofilm was formed by culturing for 72 h. The final concentrations of each food additive were 10 mM octanoic acid and 10 mM decanoic acid for medium-chain fatty acids, and 10% acesulfame K, 0.35% aspartame, 0.5% saccharin, and 7.5% sucralose for artificial sweeteners. After culturing, the biofilm was stained with 0.2% crystal violet staining solution, and the biofilm-forming ability was judged from the degree of staining (Figure 3). As a result, biofilm formation was reduced to 28% with octanoic acid and 14% with decanoic acid (Figure 3A,B). For artificial sweeteners, biofilm formation was reduced to 10% with acesulfame K and 15% with sucralose (Figure 3C,D,F).

### 2.4. Bactericidal Activity of Food Additives against Bacteria in Biofilms

**Figure 4:**
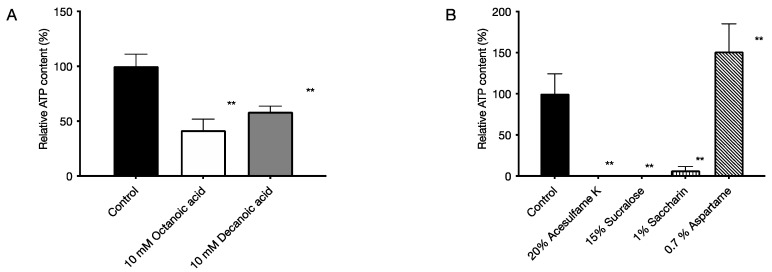
Bactericidal activity of food additives against bacteria in biofilms of *P. gingivalis*. The ATP content was measured by chemiluminescence to determine the bactericidal activity against the bacteria in the biofilm. The graph’s vertical axis shows the ATP content when the ATP content in the absence of food additives was set to 100%, and the horizontal axis shows the added food additives. (**A**) 5 mM octanoic acid and 5 mM decanoic acid, (**B**) 5% acesulfame K, 5% sucralose, 0.25% saccharin, and 0.25% aspartame. Data in the graph represent the mean ± SD. n = 3, representative data from 5 or more repeated experiments are shown. ** indicates *p* < 0.01, which is significant for ATP content in the absence of medium-chain fatty acids.

The culture solution of *P. gingivalis* was cultured for 96 h to form a biofilm, and then each food additive was added and cultured for 72 h. After culturing, the ATP content was measured to determine the bactericidal activity against the bacteria in the biofilm. As a result, the medium-chain fatty acid octanoic acid reduced the ATP content to 41% and decanoic acid to 59% (Figure 4A). The artificial sweetener acesulfame K reduced the ATP content to 0.2%, saccharin reduced the ATP content to 3%, and sucralose reduced the ATP content to 0% (Figure 4B).

### 2.5. Cytotoxic Effects on Human-Derived Cells

**Figure 5:**
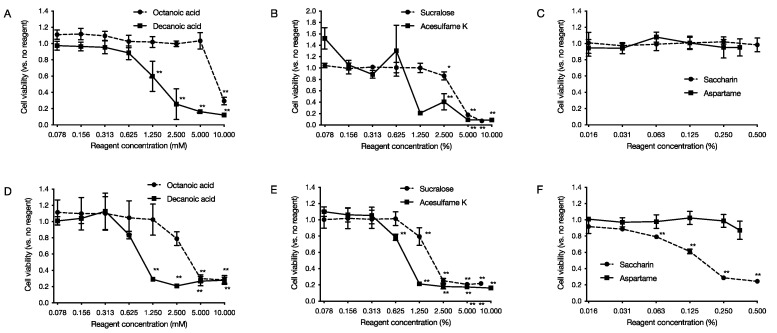
Cytotoxic effects of food additives on human-derived cells. After 24 h of incubation with limiting dilutions of food additives added to each cell, MTT reagent was added, and cytotoxicity was measured. The graph’s vertical axis shows the cell viability when the cell viability in the absence of food additives is set at 1.0. The horizontal axis shows the added concentration of food additives. (**A**–**C**) show the data of cytotoxicity against HeLa cells, and (**D**–**F**) show the data of cytotoxicity against MOLT-4 cells. Data in the graph represents the mean ± SD. n = 4 representative data of the experiment are shown. * indicates *p* < 0.05, ** indicates that each is significant at *p* < 0.01 relative to cell viability in the absence of medium-chain fatty acids.

Cytotoxicity tests on human-derived cells were carried out for six food additives, medium-chain fatty acids, and artificial sweeteners at concentrations that affected the growth of *P. gingivalis*. After adding each food additive that was limited-diluted to the culture medium of human-derived cells and culturing at 37 °C under 5% CO2 for 24 h, the MTT reagent was added to determine cell viability in the presence or absence of cytotoxicity. As a result, it was found that medium-chain fatty acids were cytotoxic in a concentration-dependent manner from 10 mM for octanoic acid to 1.25 mM for decanoic acid for HeLa cells (Figure 5A). For MOLT-4 cells, it was found that octanoic acid was cytotoxic from 5 mM and decanoic acid from 5 mM in a concentration-dependent manner (Figure 5D). Regarding the artificial sweetener, it was found that acesulfame K was cytotoxic to HeLa cells from 2.5% and sucralose from 2.5% in a concentration-dependent manner (Figure 5B,C). For MOLT-4 cells, acesulfame K showed cytotoxicity from 0.625%, saccharin from 0.125%, and sucralose from 1.24% in a concentration-dependent manner (Figure 5E,F).

## 3. Discussion

In this study, we used *P. gingivalis*, a pathogenic bacterium of chronic periodontitis, to evaluate the effects of six food additives (octanoic acid, decanoic acid, acesulfame K, aspartame, saccharin, and sucralose) used in foods for the elderly on the growth and biofilm formation of *P. gingivalis*. The effects of the six food additives (octanoic acid, decanoic acid, acesulfame K, aspartame, saccharin, and sucralose) on *P. gingivalis* growth and biofilm formation were evaluated in vitro. The results showed that all six food additives showed growth inhibitory (Figure 1) and bactericidal activity against *P. gingivalis* (Figure 2). In addition, octanoic acid, decanoic acid, acesulfame K, and sucralose inhibited biofilm formation by *P. gingivalis* at concentrations that inhibited growth (Figure 3). In addition, octanoic acid, decanoic acid, acesulfame K, saccharin, and sucralose showed bactericidal activity against *P. gingivalis* in biofilms at concentrations that inhibited growth (Figure 4). On the other hand, the cytotoxicity to human cells (HeLa, MOLT-4) of the concentrations that showed growth inhibitory effects on periodontopathogenic bacteria was examined. Octanoic acid, decanoic acid, acesulfame K, and saccharin showed cytotoxicity, while aspartame showed no cytotoxicity (Figure 5).

Currently, some of the digestive nutrition products in use contain 3.3% medium-chain fatty acids (Nestle Health Science, Pepatamen, <https://www.nestlehealthscience-me.com/en/brands/peptamen/peptamen>. accessed on 11 November 2021). In the present study, the medium-chain fatty acids octanoic acid and decanoic acid showed growth inhibitory effects at 5 mM (0.072%) and 1.25 mM (0.0215%), respectively. Therefore, it is possible that the concentrations of these compounds actually in use may have an effective growth inhibitory effect on *P. gingivalis*. As for artificial sweeteners, the concentrations in the foods in which they are used are not listed and are unknown, but the minimum concentrations that can be used are 0.05% (500 mg/kg) for acesulfame K, 0.1% (1000 mg/kg) for aspartame, 0.02% (200 mg/kg) for saccharin, and 0.04% (400 mg/kg) for sucralose according to the Codex General Standard for Food Additives (Codex Alimentarius international food standard, Codex General Standard for Food Additives (GSFA) Online Database, https://www.fao.org/fao-who-codexalimentarius/codex-texts/dbs/gsfa/pt/. accessed 11 November 2021) [25] on dietetic foods intended for special medical purpose. The results of this study showed that acesulfame K, aspartame, saccharin, and sucralose significantly inhibited growth at 1.25%, 0.0625%, 0.03125%, and 0.625%, respectively, indicating that only aspartame has a growth inhibitory effect at concentrations that can be used in practice. Therefore, only aspartame has a growth inhibitory effect at concentrations that can be used in practice.

On the other hand, the 50% inhibition of bacterial growth (IC_50_) and 50% cytotoxicity against human-derived cells (CD_50_) of the food additives used in this study were determined as follows: IC_50_ of octanoic acid and decanoic acid were 7.12 mM and 0.910 mM. That of acesulfame K, aspartame, saccharin, and sucralose were respectively 1.03%, 0.0744%, 0.0445%, and 0.921%. CD_50_ for HeLa was 8.17 mM for octanoic acid, 1.52 mM for decanoic acid, >0.5% for aspartame, >0.35% for saccharin, and 3.61% for sucralose. Acesulfame K could not be calculated due to large variability. The CD_50_ values for MOLT-4 were 3.77 mM for octanoic acid, 0.958 mM for decanoic acid, 0.883% for acesulfame K, >0.5% for aspartame, 0.159% for saccharin, and 1.82% for sucralose. The CD_50_/IC_50_ ratio of each substance was calculated as follows: octanoic acid, 1.15 (HeLa), 0.529 (MOLT-4); decanoic acid, 1.67 (HeLa), 1.05 (MOLT-4); acesulfame K, 0.809 (MOLT-4); aspartame, >6.72 (HeLa), >6.72 (MOLT-4), aspartame > 6.72 (HeLa); saccharin > 7.87 (HeLa), 3.57 (MOLT-4), and sucralose > 3.92 (HeLa), 1.98 (MOLT-4), suggesting that aspartame and saccharin are relatively useful.

In addition, it is necessary to act rapidly in the oral cavity since periodontopathogenic bacteria are present in the oral cavity. All the food additives used in this study showed bactericidal activity, but no food additive reduced the viable count of *P. gingivalis* to zero at 0 h of action. We think, for instance, that highly viscous foods or chewable tablets with these food additives can exist in the oral cavity for a certain period. If the retention time within the oral cavity is prolonged, it is considered that these food additives would be easy to reach the inside of the pocket. At the same time, it is necessary to have an inhibitory effect on biofilm formation and a bactericidal effect on the bacteria in the biofilm since periodontopathogenic bacteria exist in the biofilm called dental plaque. The results showed that octanoic acid, decanoic acid, acesulfame K, and sucralose effectively inhibited biofilm formation at concentrations that inhibited growth and showed bactericidal activity against bacteria in the biofilm. Electron microscopic analysis of the biofilm morphology showed that acesulfame K, saccharin, and sucralose did not change the morphology of the artificial sweeteners used in this study (unpublished results), suggesting that they may not act on biofilms.

The mechanism of action described above is not clear. In general, possible mechanisms of bactericidal activity against bacteria include inhibition of cell wall synthesis, damage to cell membranes, inhibition of nucleic acid synthesis, inhibition of protein synthesis pathways, and inhibition of metabolic pathways [26]. The above mechanism of action can be evaluated by transmission electron microscopy, measurement of the uptake rate of nucleic acids and amino acids containing radioisotopes, metabolomic analysis, and gene expression analysis, and will be analyzed in the future. As for artificial sweeteners, since *P. gingivalis* cannot utilize sugar as a nutrient source [27], it is unlikely that they have bactericidal effects on sugar metabolism pathways.

Overall, the results of this study suggest that decanoic acid and aspartame are effective in preventing periodontal disease. Decanoic acid was superior in inhibiting the growth of *P. gingivalis* at the actual concentration used, inhibiting biofilm formation, and showing bactericidal activity against bacteria in the biofilm. The CD_50_/IC_50_ ratios were 1.67 (HeLa) and 1.05 (MOLT-4), relatively low. Aspartame inhibited the growth of *P. gingivalis* at practically usable concentrations, showed no cytotoxicity to human-derived cells at similar concentrations, and had a CD_50_/IC_50_ ratio of 6.72 or higher for both cells. However, there are some disadvantages, such as inhibiting biofilm formation and the lack of bactericidal activity against *P. gingivalis* in biofilms. To solve these problems, we would like to study the effect of the combination of two or more food additives on *P. gingivalis*. The mixture may have a synergistic effect on the biofilm and may affect *P. gingivalis* at low concentrations without cytotoxicity.

This study examined only *P. gingivalis*, a keystone pathogen of periodontal disease. However, the oral cavity hosts a plethora of tissues that serve various functions, and bacteria, including periodontal pathogens other than *P. gingivalis*, viruses, and fungi, live in a subtly balanced ecosystem in this complex environment [28]. Lifestyle-related factors, such as aging, stress, smoking, alcohol consumption, and diabetes, affect the alterations of oral conditions, such as salivary hypofunction [18]. Further, the oral cavity and gut are anatomically connected, and changes in each environment, such as innate immunity between microbiome and mucosa, can affect the structures of both microbiomes that colonize mucosal surfaces in the digestive systems in a bidirectional way [29]. The antibacterial activity of these six food additives has not yet been checked in the polybacterial condition or the co-existence of gingival cells. Next, these experiments should be performed. If other periodontal bacteria show susceptivity with food additives, the possibility of their preventive effect on periodontal disease will increase. In addition, the present study was conducted in vitro. We should further investigate the effect of food additives on the prevention, treatment, and progression of periodontal disease in vivo using a mouse periodontal disease model. Moreover, intervention experiments of these food additives in humans are necessary for future study.

It has been reported that food choice significantly influences the prevention of periodontal diseases [21,30]. A previous report showed that artificial sweeteners used in many foods have antibacterial effects against *P. gingivalis* and *A. actinomycetemcomitans* [24]. However, the effects of food additives in foods for the elderly and foods for adjusted swallowing on periodontal disease are largely unknown. Our in vitro results of this study cannot be extrapolated directly to in vivo or clinical practice. However, the food additives shown in the study are used in foods for people with dysphagia, who are considered to be at high risk of aspiration pneumonia. These findings may help clarify the effects of food additives on periodontopathogenic bacteria.

## 4. Materials and Methods

### 4.1. Bacterial Culture

All experiments were performed using the type strain *P. gingivalis* 33277. *P. gingivalis* was cultured in tryptic soy broth (TSB) broth containing Brain-Heart Infusion (BD Biosciences Franklin Lakes, NJ, USA), 5 μg/mL Hemin (Nacalai tesque, Kyoto, Japan), 1 μg/mL Menadione (Nacalai tesque, Kyoto, Japan), or on the TSB agar supplemented with agar to a final concentration of 15 g/L at 37 °C under the anaerobic condition in the square jar with Anaeropac Kenki (Mitsubishi Gas Chemical, Tokyo, Japan).

### 4.2. Food Additives

Before the experiment, we investigated and listed food additives used in commercially available foods ranging from general foods to foods for the elderly with swallowing adjustments from food labeling and the manufacturer’s website, such as Meiji (https://www.meiji.com/global/, accessed on 11 November 2021) and Nestle (https://www.nestle.com/brands/healthcarenutrition, accessed on 11 November 2021). Then, we selected six additives that are used relatively frequently from the list and have been very few reports on *P. gingivalis*. In this study, medium-chain fatty acids (octanoic acid, decanoic acid) (Tokyo Chemical Industry, Tokyo, Japan) and artificial sweeteners (acesulfame K, aspartame, saccharin, sucralose) (Tokyo Chemical Industry, Tokyo, Japan), which were commercially available as reagents, were examined. Medium-chain fatty acids were dissolved in DMSO (Nacalai tesque, Kyoto, Japan), and the sweeteners were dissolved in sterile water. The examined concentrations of these food additives were determined based on previous reports [24,31,32,33,34].

### 4.3. Determination of Minimum Inhibitory Concentration (MIC)

After anaerobic culture on the TSB agar medium, *P. gingivalis* was suspended in the TSB broth so that the absorbance at 600 nm of the bacterial suspension was adjusted to 0.1. One hundred microliters of the bacterial suspension were seeded to a 96-well plate, and 100 μL of TSB broth containing each food additive with limiting dilutions was added. After 48 h of incubation under anaerobic conditions by Anaeropack Kenki (Mitsubishi Gas Chemical, Tokyo, Japan), the absorbance at 600 nm was measured using a plate reader (Bio-Rad model 680, Bio-Rad Laboratories, Hercules, CA, USA). The growth of bacteria was judged, and MIC was determined by the absorbance of the bacterial culture [31].

### 4.4. Time-Kill Assay

Suspension of *P. gingivalis* adjusted to a concentration of 1 × 10^8^ CFU/mL in TSB broth was mixed with each food additive in a microtube. After incubation for 2 h, 4 h, 8 h, and 24 h under anaerobic conditions, the bacteria were recovered by spinning down, washed with phosphate buffered saline (PBS), resuspended, diluted to the limit, applied to TSB agar medium, and incubated under anaerobic conditions for 10 days. The bactericidal activity was determined by the number of bacterial colonies formed on the agar [35].

### 4.5. Inhibition of Biofilm Formation

*P. gingivalis* was seeded at 1 × 10^9^ CFU/mL in a 24-well plate, and the food additives diluted to each concentration were added. Biofilm was formed by culturing for 72 h under anaerobic conditions. After removing the culture medium, the biofilm was washed 3 times with PBS. Then 0.5 mL of 0.2% crystal violet solution was added and incubated at 37 °C for 1 min to stain the biofilm. After removing the staining solution and washing 3 times with PBS, 0.5 mL of 100% ethanol was added for decolorization. The absorbance of the decolorization solution was measured at 595 nm, and the biofilm-forming ability in the presence of food additives was determined from the absorbance [36,37].

### 4.6. Bactericidal Test against Bacteria in Biofilm

*P. gingivalis* (6 × 10^8^ CFU/mL) was seeded in a 96-well plate, and a biofilm was formed under anaerobic conditions for 96 h. One hundred microliters of food additives at various concentrations in TSB broth were added to the well and further incubated for 72 h under anaerobic conditions. After the culture medium was removed, the well was washed 3 times with distilled water. Then, BacTiter-Glo ™ (Promega, Madison, WI, USA) was added, and the intensity of chemiluminescence was measured using Multimode Detector DTX800 (BECKMAN COULTER, Brea, CA, USA). The ATP content was measured by the intensity of the chemiluminescence, and the bactericidal activity of the food additive against the bacteria in the biofilm [36].

### 4.7. Cytotoxicity Test

Commercially available HeLa cells, derived from human cervical adenocarcinoma [38], and MOLT-4 cells, derived from human T-cell acute lymphoblastic leukemia [39], were used because these cell lines are generally used for the cytotoxicity test [40,41]. Cells were prepared to 5 × 10^5^ cells/mL using DMEM or RPMI with 10% fetal bovine serum (FBS), respectively, and seeded into a 96-well plate at 100 μL each. After incubation at 37 °C for 24 h under 5% CO_2_, each food additive was added at a limiting dilution using the medium and incubated for another 24 h. Then, 10 μL of MTT reagent (WST-8; Nacalai Tesque, Kyoto, Japan) was added to each well and incubated at 37 °C for 2 h under 5% CO_2_. Finally, the absorbance at 450 nm (reference wavelength 630 nm) was measured by a plate reader. The cell viability was determined by the absorbance value [42].

### 4.8. Statistical Analysis

The results of the MIC test, the viable cell count test, the bactericidal test against the bacteria in the biofilm, and the cytotoxicity test were analyzed by one-way analysis of variance, and the Dunnett method was used for multiple comparisons. The results of the biofilm formation inhibition test were analyzed by an unpaired t-test.

## Data Availability

Not applicable.

## Supplementary Materials

The following are available online at https://www.mdpi.com/article/10.3390/pathogens11010065/s1, Figure S1. Growth inhibition effect of Medium-chain fatty acids (A) and artificial sweeteners (B) on *P. gingivalis* TCD60 and W83.
