# Supplementary material for: Analysis of the Effects of Food Additives on Porphyromonas gingivalis
Source: Pathogens. 2022 Jan 4;11(1):65. doi: 10.3390/pathogens11010065 (PMC8779409; doi:10.3390/pathogens11010065)
Supplement: Supplementary file 1 [file pathogens-11-00065-s001.zip › pathogens-1495206-supplementary.pdf]

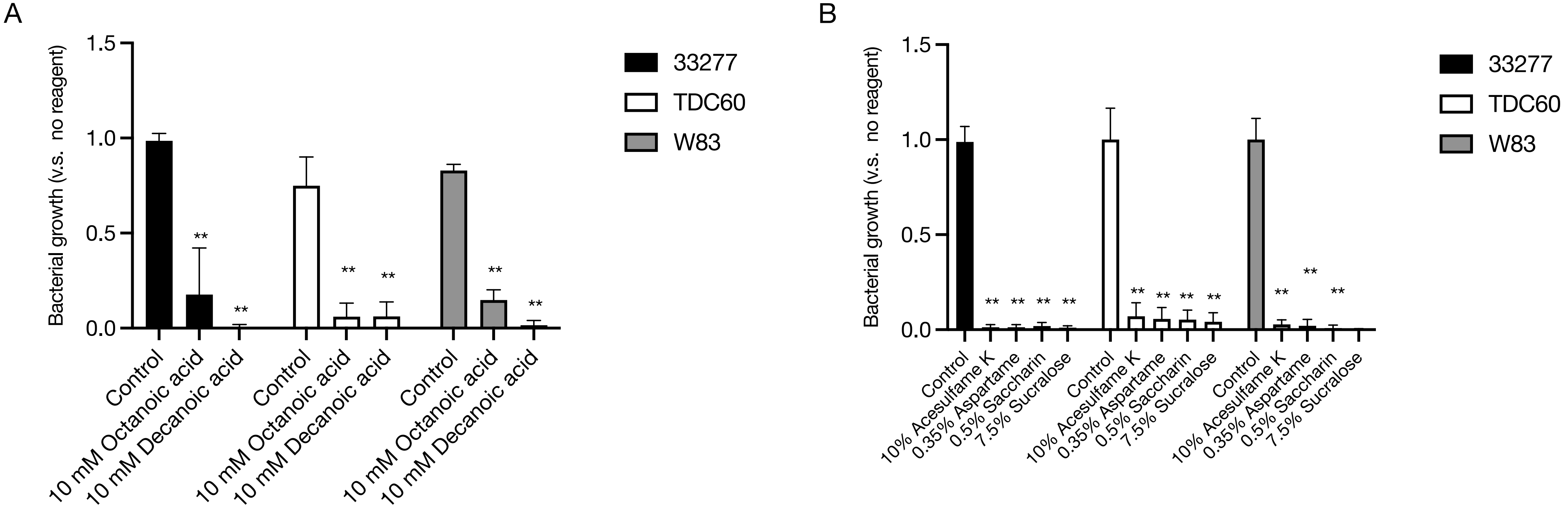

Figure S1. Growth inhibition effect of Medium-chain fatty acids (A) and artificial sweeteners (B) on *P. gingivalis* TCD60 and W83. ** indicates that each is significant at $p < 0.01$.
